# Prognostic Role of MicroRNA 222 in Patients with Glioma: A Meta-analysis

**DOI:** 10.1155/2020/4689689

**Published:** 2020-09-25

**Authors:** Yanlin Song, Jing Zhang, Min He, Jianguo Xu

**Affiliations:** ^1^Department of Neurosurgery and National Clinical Research Center for Geriatrics, West China Hospital, Sichuan University, Chengdu, China; ^2^Department of Biotherapy, Cancer Center, West China Hospital, Sichuan University, Chengdu, China; ^3^Department of Critical Care Medicine, West China Hospital, Sichuan University, Chengdu, China

## Abstract

**Background:**

Several studies have focused on the prognostic role of microRNA 222 in glioma. But different conclusions were drawn by these studies. We aimed to systematically evaluate the role of microRNA 222 in glioma by conducting a meta-analysis.

**Methods:**

A systematic literature search until January 2020 was conducted in Web of Science, EMBASE, Cochrane Library, PubMed, and China National Knowledge Infrastructure. The general characteristics and relevant data of nine articles were extracted. Hazard ratios (HRs) with 95% confidence intervals (CIs) were applied to evaluate the prognostic role of microRNA 222 in glioma. The primary outcomes were overall survival (OS) and disease-free survival (DFS).

**Results:**

Nine articles (11 data sets) with 1564 patients were included. We systematically evaluated the role of microRNA 222 for OS and DFS in glioma patients (HR for OS = 1.72; 95% CI, 1.31-2.26; *p* = 0.001; HR for DFS = 1.02; 95% CI, 0.86-1.22; *p* = 0.032). Subgroup analyses were performed according to the sources of patients, the types of the samples, the stages of the tumors, the methods for detecting the microRNA 222, and the sample size. No significant publication bias was found.

**Conclusion:**

In conclusion, our study provided evidence that a high expression of microRNA 222 was related to worse overall survival in glioma patients. However, given the limited study number, more high-quality studies are warranted in the future.

## 1. Introduction

Glioma is the most common cancer in the central nervous system with high mortality and recurrence [1]. Although significant advances have been achieved in the treatment, the prognosis of glioma patients is still very poor [2]. So, it is urgent to explore a novel biomarker to predict the prognosis of glioma.

MicroRNAs are a class of single-strand RNAs which silence the genes by targeting the mRNAs [3]. It is proven that aberrant expressions of microRNAs are related to the proliferation, migration, and invasion of tumor cells [4–6]. In addition, some microRNAs have been proved to play oncogenic roles [7–12]. Therefore, many studies focused on microRNAs. For example, microRNA 373, 15, 107, 133, and 211 were reported to be related to the prognosis of glioma [13–17].

MicroRNA 222 is reported to be related to the proliferation, invasion, and migration of several types of tumor cells [18–20]. Downregulation of microRNA 222 inhibited the growth and angiogenesis of glioma and sensitized glioma cells to temozolomide according to the previous studies [21–23]. Moreover, a high level of microRNA 222 was proven to promote the proliferation, invasion, migration, angiogenesis, radioresistance, and chemoresistance of glioma cells [24–27]. Considering the relationship between microRNA 222 and glioma, several studies investigated the role of microRNA 222 on the prognosis of glioma [11, 17, 26, 28–33]. But different conclusions were drawn by these studies. In order to reach an agreement, we evaluated the prognostic role of microRNA 222 in glioma patients by conducting a meta-analysis.

## 2. Method

### 2.1. Search Strategy

A systematic literature search until January 2020, was conducted in Web of Science, EMBASE, Cochrane Library, PubMed, and China National Knowledge Infrastructure. The key words, microRNA 222 (microRNA 222-3p or hsa-miR-222), glioma (astrocytoma or glioblastoma or ependymoma or subependymal or ganglioglioma or gliosarcoma or medulloblastoma or oligodendroglioma), and prognosis (survival) and all possible combinations were included. Moreover, the article was organized based on the Preferred Reporting Items for Systematic Reviews and Meta-Analyses (PRISMA) checklist (see available here).

### 2.2. Inclusion and Exclusion Criteria

All randomized control trials (RCT) and cohort studies were included in our study. Included studies met the following inclusion criteria: (1) the diagnosis of glioma was proven by pathological examination, (2) the prognostic role of microRNA 222 on glioma was studied in the study, and (3) hazard ratio (HR) and 95% CI were shown or can be calculated in the article. In addition, the studies that met the following criteria were excluded: (1) reviews, letters, case reports, and opinions from experts; (2) no available data for HR was shown in the article; and (3) duplicate data or figures.

### 2.3. Data Extraction

The general information, HR, and 95% CI were extracted from the selected articles. The general information included the name of author, the country of study, type of sample, number of samples, stage of glioma, the cut-off value, and the methods for detecting microRNA 222. The data which can be used to calculate the HR was also extracted according to Tierney's report [34]. If multivariate analyses and univariate analyses were both applied to calculate HR, the results of multivariate analyses were extracted.

### 2.4. Statistical Analysis

The Log[HR] and stand error (SE) calculated from pooled HRs with 95% CIs were used to analyze the association between the expression of microRNA 222 and survival of glioma. The primary outcomes were overall survival (OS) and disease-free survival (DFS). Review Manager 5.3.5 (Cochrane Collaboration, Oxford, UK) was used to calculate Log[HR] and SE from HRs and *p* values. HRs and *p* values were not available in studies performed by Li and Zhang; Log[HR] and SE were extracted from the KM curve by Engauge Digitizer 10.0 (free software downloaded from https://sourceforge.net/projects/digitizer/). A random effects model was applied to calculate the pooled HR whether the heterogeneity was significant (*I*^2^ > 50% or *p* < 0.1). Subgroup analyses were performed according to the sources of patients, the types of the samples, the stages of the tumors, and the methods for detecting the microRNA 222. The stability of our study was assessed with sensitivity analysis. The pooled HR was calculated by STATA 11.0 (StataCorp, College Station, TX, USA). The publication bias was analyzed by Begg's test.

## 3. Results

### 3.1. Study Selection

**Figure 1:**
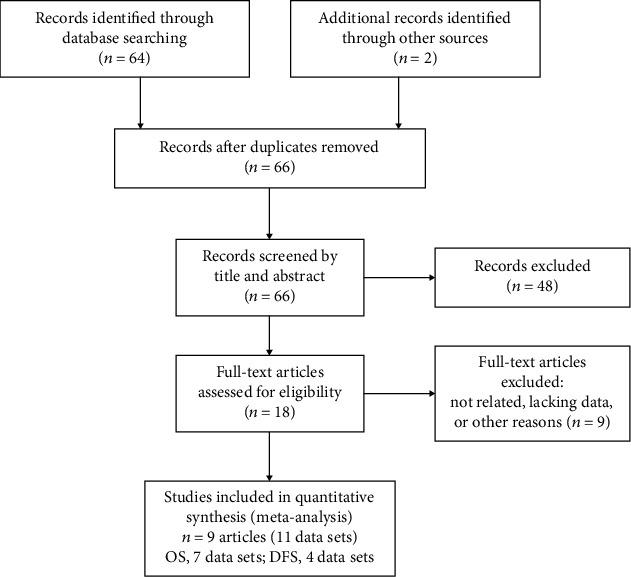
Selection process of studies.

As shown in Figure 1, the studies were selected following the PRISMA flow diagram (see available here). At the end, 64 articles by literature search and 2 articles found by other sources were included in the first round of research. No duplicated articles were found. After that, 48 unrelated articles were excluded by screening the title and abstract of these articles. At last, the full text of the remaining18 articles were carefully screened and only 9 articles (11 data sets) were included in our study [11, 26, 28–33, 35]. Among these studies, 7 data sets were related to overall survival (OS) and 4 data sets were related to the disease-free survival (DFS). All of the data sets were evaluated by the Newcastle–Ottawa scale criteria.

### 3.2. Basic Characteristics of the Included Studies

The basic characteristics of the included studies were presented in Table 1. A total of 1564 patients were included in these studies from 2011 to 2018. The patients were from China or the USA. The samples which were used for detecting microRNA 222 were mostly from tumor tissues. The cut-off value and methods for detecting microRNA 222 were also extracted from these studies. The median value of the microRNA 222 level was commonly defined as the cut-off value according to the included studies.

### 3.3. Overall Survival and Disease-Free Survival

**Figure 2:**
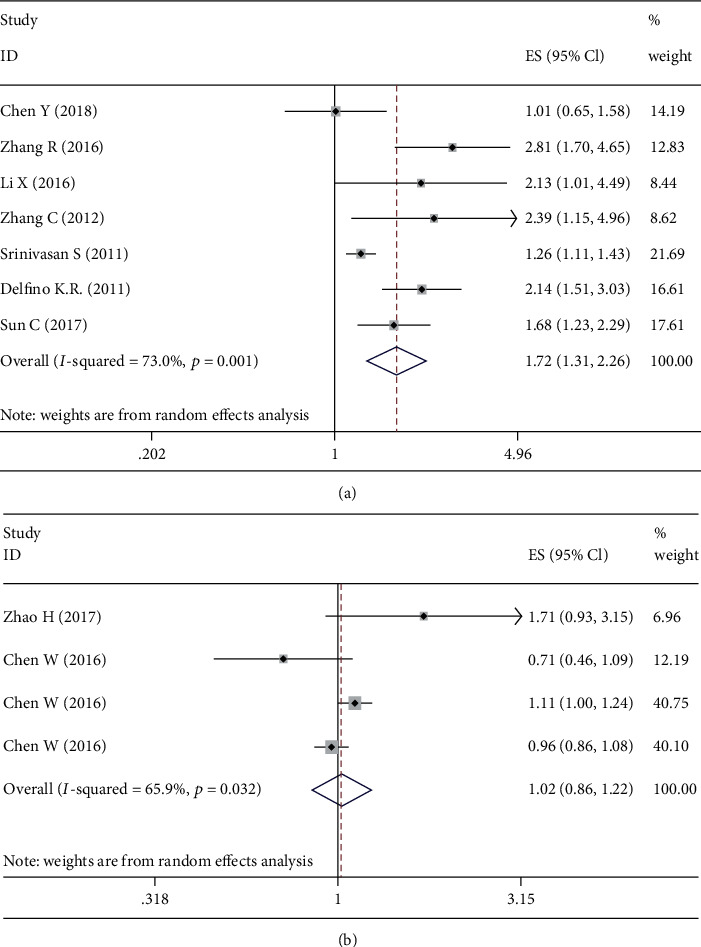
Pooled hazard ratio of higher microRNA 222 for overall survival (a) and disease-free survival (b) in patients with glioma.

The pooled HR for OS was provided by 7 data sets. As shown in Figure 2(a), a high expression of microRNA 222 was related to worse overall survival (HR = 1.72; 95% CI, 1.31-2.26; *p* = 0.001). But the HR for DFS implied that the high expression of microRNA 222 might not be related to the worse disease-free survival (HR = 1.02; 95% CI, 0.86-1.22; *p* = 0.032; Figure 2(b)). In order to explore the stability of our results, sensitivity analyses were performed by deleting one study at a time and recalculating the pooled HR at the same time. As expected, the recalculated HRs still proved that the high expression of microRNA 222 was related to the poor prognosis of glioma (Table 2).

### 3.4. Subgroup Analysis for Overall Survival

To further explore the effects of other factors, like sources of patients, types of samples, tumor grades, and methods for detecting microRNA 222, on the prognosis of glioma patients, we performed subgroup analysis.

#### 3.4.1. Sources of Patients

Among the 7 data sets, 4 data sets were performed in China and 3 data sets were performed in the USA (Table 3). The pooled HR of the China and USA groups were 1.90 (95% CI, 1.10-3.26; *p* = 0.017) and 1.61 (95% CI, 1.16-2.22; *p* = 0.008), respectively, which indicated that the high expression of microRNA 222 was related the poor prognosis of glioma both in China and the USA.

#### 3.4.2. Types of Samples

Only 1 data set tested the microRNA 222 through the blood of patients. So, we calculated the pooled HR by excluding this study (Table 3). The pooled HR changed slightly to 1.58 (95% CI, 1.22-2.04; *p* = 0.011), which further proved the role of microRNA 222 on glioma.

#### 3.4.3. Tumor Grades

The tumor grade was also a potential factor for the prognosis of glioma. Four data sets only included stage IV patients of glioma while 3 data sets included stage I-IV patients of glioma (Table 3). So, the pooled HR of these studies were analyzed, respectively. As expected, the high expression of microRNA 222 was still related to the poor prognosis of glioma according to the pooled HR of the different stage patients (HR of IV stage patients = 1.47; 95% CI, 1.11-1.94; *p* = 0.01; HR of I − IV stage patients = 2.53; 95% CI, 1.76-3.63; *p* = 0.82).

#### 3.4.4. Methods for Detecting MicroRNA 222

Three data sets detected the microRNA 222 by Q-PCR and 1 data set by immunohistochemistry scoring. The remaining 3 data sets did not mention the method for detecting the microRNA 222. So, we calculated the pooled HR of the Q-PCR group (HR = 1.79; 95% CI, 0.90-3.57; *p* = 0.009).

#### 3.4.5. Sample Size

The pooled HR of three data sets which included patients less than 100 was 2.53 (95% CI, 1.76-3.63; *p* = 0.82). As to the studies which included patients more than 100, the pooled HR was 1.47 (95% CI, 1.12-1.94; *p* = 0.007).

### 3.5. Publication Bias

**Figure 3:**
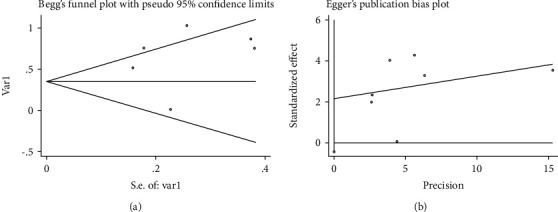
Begg's (a) and Egger's(b) publication bias plot of the included studies.

The publication bias were evaluated by Begg's test (*p* = 0.548) and Egger's test (*p* = 0.085). The funnel plot was shown in Figure 3. No obvious publication bias was presented in these studies.

## 4. Discussion

MicroRNA 222 which acts as a good biomarker for the prognosis of patients is found in several cancers [17, 36, 37]. Numbers of clinical studies have explored the relationship between microRNA 222 and prognosis of glioma patients [11, 17, 26, 28–32, 38–45]. But different conclusions were drawn by these studies. To reach an agreement, we conducted a systematic meta-analysis. In this study, the pooled HR was calculated from the extracted data (HR for OS = 1.72; 95% CI, 1.31-2.26; HR for DFS = 1.02 95% CI, 0.86-1.22). It indicated that a high expression of microRNA 222 predicted the worse overall survival other than the recurrence or progression in glioma. But only four data sets were used for the analysis of DFS in our study. In addition, three data sets were from the same study. So, more studies focusing on DFS are needed to draw a convincing conclusion.

In order to explore the stability of our results, sensitivity analyses were performed by deleting one study at a time and recalculating the pooled HR at the same time. As expected, the recalculated HRs still proved that the high expression of microRNA 222 was related to the poor prognosis of glioma (Table 2). We then explored the potential role of the sources of patients, types of samples, tumor grades, methods for detecting microRNA 222, and sample size by subgroup analysis. Finally, the publication bias was analyzed by Begg's and Egger's tests. All of the subsequent analyses further proved the role of microRNA 222 in glioma.

Glioma characterized with rapid proliferation, high malignancy, and resistant to conventional therapy was reported to have poor prognosis in previous studies [46]. Intriguingly, microRNA 222 was proven to be related to the proliferation, migration, and invasion of glioma cells [18–20]. An obvious activation of AKT was found in microRNA 222 overexpression glioma cells. And the significant changes of AKT related genes were also observed in these cells [27]. Besides, PTP_*μ*_ was found as a new target in this process [25]. An inverse correlation has been observed between PTP_*μ*_ and microRNA 222 in vivo and in vitro. MicroRNA 222 promoted the migration and proliferation of glioma cells with downexpression of PTP_*μ*_. And the reexpression of PTP_*μ*_ was able to revert the effects. The overexpression of microRNA 222 was also verified to be associated with radioresisitance and chemoresistance [21, 24]. MicroRNA 222 was identified as a key molecular in temozolomide-resistant glioma patients, and knockdown of microRNA 222 sensitized glioma cells to temozolomide by regulating the expression of apoptosis-independent p53. Another study revealed that microRNA 222 was mediated in the radio-induced DNA damage repair, which implied the potential of microRNA 222 on the increasing radiosensitivity of glioma cells. The results mentioned above showed potential reasons why microRNA 222 might be used as a good marker for predicting the prognosis of glioma patients.

Although our results were robust, some limitations of the issue should be emphasized. Firstly, the patients included in our study only from China and the USA limited the representativeness of the prognostic role in the world. Secondly, significant heterogeneity was observed in this study. Sensitivity analysis and subgroup analysis did not eliminate heterogeneity, which might be due to the different characteristics of included patients across the studies. The limitations implied the need for more studies that focused on microRNA 222 and glioma.

In conclusion, our study provided evidence that a high expression of microRNA 222 was related to worse overall survival of glioma patients. However, given the limited study number, more high-quality studies are warranted in the future.

## Figures and Tables

**Table 1:** Characteristics of the included articles.

Author	Country	Sample	Number	Stage	Cut-off	Method	Results	HR (95% CI)	*p* value	Quality score (NOS)
Chen Y	China	Tissue	114	IV	None	Q-PCR	OS	1.01 (0.65-1.58)	0.965	9
Zhang R	China	Blood	51	I-IV	None	Q-PCR	OS	2.81 (1.70-4.65)	0.0001	8
Li X	China	Tissue	45	I-IV	Mean	Q-PCR	OS	2.13 (1.01-4.48)	0.043	8
Zhang C	China	Tissue	36	I-IV	—	IHC	OS	2.39 (1.15-4.96)	0.02	8
Sun C	USA	Tissue	548	IV	Median	—	OS	1.68 (1.23-2.29)	0.001	8
Srinivasan S	USA	Tissue	111	IV	60%	—	OS	1.26 (1.11-1.43)	0.0004	7
Delfino K.R.	USA	Tissue	253	IV	—	—	OS	2.14 (1.51-3.03)	<0.0001	7
Zhao H	USA	Blood	106	IV	Median	—	DFS	1.71 (1.07-3.63)	0.038	7
Chen W	USA	Tissue	89	IV	Median	—	DFS	0.71 (0.46-1.09)	0.12	7
			102	IV	Median	—	DFS	1.11 (0.99-1.23)	0.07	7
			109	IV	Median	—	DFS	0.96 (0.86-1.08)	0.53	7

HR: hazard ratio; CI: confidence interval; Q-PCR: quantitative polymerase chain reaction; IHC: immunohistochemistry; NOS: Newcastle–Ottawa scale.

**Table 2:** Sensitivity analysis.

Excluding article	*I* ^2^, *p*	Pooled HR (95% CI)
Chen Y	74.7%, 0.001	1.88 (1.39-2.54)
Zhang R	66.5%, 0.011	1.58 (1.22-2.04)
Li X	76.3%, 0.001	1.69 (1.26-2.25)
Zhang C	75.4%, 0.001	1.67 (1.25-2.21)
Srinivasan S	56.1%, <0.001	1.87 (1.40-2.49)
Delfino K.R.	69.7%, 0.006	1.64 (1.22-2.19)
Sun C	76.2%, 0.001	1.75 (1.25-2.45)

HR: hazard ratio; CI: confidence interval.

**Table 3:** Summary of meta-analysis results.

Stratified study	Data sets	Pooled HR (95% CI)	*p* value	Heterogeneity	
				*I* ^2^	*p* value
OS	7	1.72 (1.31-2.26)	<0.001	73.00%	0.001
DFS	4	1.02 (0.86-1.22)	0.807	65.90%	0.032
Country					
China	4	1.90 (1.10-3.26)	0.021	70.70%	0.017
USA	3	1.61 (1.16-2.22)	0.004	79.20%	0.008
Material					
Tissue	6	1.58 (1.22-2.04)	0.001	66.50%	0.011
Blood	1	2.81 (1.7-4.65)	0.0001	—	—
Stage					
IV	4	1.47 (1.11-1.94)	0.007	73.70%	0.01
I-IV	3	2.53 (1.76-3.63)	<0.001	0.00%	0.82
Method					
Q-PCR	3	1.79 (0.90-3.57)	0.099	78.80%	0.009
Immunohistochemistry scoring	1	2.39 (1.15-4.96)	0.02	—	—
Sample size					
>100	4	1.47 (1.12-1.94)	0.01	73.70%	0.007
<100	3	2.53 (1.76-3.63)	<0.001	0.00%	0.82

HR: hazard ratio; CI: confidence interval; Q-PCR: quantitative polymerase chain reaction; IHC: immunohistochemistry.

## Supplementary Materials

Supplementary Materials Figure 1: PRISMA 2009 flowchart. Table: PRISMA checklist.
