# Supplementary material for: Prognostic Role of MicroRNA 222 in Patients with Glioma: A Meta-analysis
Source: Biomed Res Int. 2020 Sep 25;2020:4689689. doi: 10.1155/2020/4689689 (PMC7533000; doi:10.1155/2020/4689689)
Supplement: Supplementary Materials — Figure 1: PRISMA 2009 flowchart. Table: PRISMA checklist. [file 4689689.f1.zip › PRISMA Flow Diagram.doc]

**PRISMA 2009 Flow Diagram**

**Screening**

**Included**

**Eligibility**

**Identification**

Records identified through database searching
(n = 64)

Additional records identified through other sources
(n = 2)

Records after duplicates removed
(n = 66)

Records screened
(n = 66)

Records excluded
(n = 48)

Full-text articles assessed for eligibility
(n = 18)

Full-text articles excluded:

not related, lacking data, or other reasons (n = 9)

Studies included in qualitative synthesis
(n = 9)

Studies included in quantitative synthesis (meta-analysis)
(n = 9)
